# Editorial: Insights in bone research: 2022

**DOI:** 10.3389/fendo.2023.1292931

**Published:** 2023-10-03

**Authors:** Giacomina Brunetti, Jonathan H. Tobias

**Affiliations:** ^1^ Department of Biosciences, Biotechnologies and Environment, University of Bari Aldo Moro, Bari, Italy; ^2^ Musculoskeletal Research Unit, Translational Health Sciences, Bristol Medical School, University of Bristol, Bristol, United Kingdom

**Keywords:** osteocyte, bone cell, osteosarcoma, fragility fracture, osteoimmunology, organoid

Bone research is continuously updated with the discovery of new mechanisms regulating bone cell activity and thus bone remodeling. Osteoblasts are the bone forming cells and their activity is linked to that of osteoclasts, the bone resorbing cells. However, the cells that orchestrate their balanced function are the osteocytes, representing the most numerous cells in this tissue embedded in the matrix. In pathological conditions the equilibrated activity of bone cells is altered and thus alteration of bone quality and structure arise, leading to the development of bone diseases, such as osteoporosis, fractures, etc. The activity of bone cells can be modulated by different stimuli and due to the necessity to test potential new therapeutic targets 3D cultures as well as bone organoids have been realized.

Marahleh et al. wrote an interesting review on osteocytes and their relationship with osteoclasts. It is known that the differentiation of osteoblasts into osteocytes is associated with different morphological and functional changes. The authors describe how osteocytogenesis is associated with molecular and morphological changes and organization of the osteocytic lacuna-canalicular network. Using knowledge from transcriptomic analyses of osteocytes they discuss the regulatory role of osteocytes in promoting osteoclastogenesis with an emphasis on the case of osteoclastogenesis in models of anosteocytic bones. The latter is used to study the role of osteocytes in modulating bone cell activity, and is typical of some vertebrate groups, such as modern teleosts, a group representing half vertebrate and bony fish (1). The authors concluded that osteocytes exhibit several redundant means of stimulating osteoclastogenesis, as well as illustrating the difficulty in studying their role using different *in vitro* and *in vivo* models.

This limitation led Knowles et al. to develop a new model to study osteocyte activity and test therapeutic targets. The authors developed a primary human 3D organotypic culture system that mimics the formation of mature osteocytes in bone. In this model primary human osteoblasts were seeded in a fibrinogen/thrombin gel around 3D-printed hanging posts. The organoids were viable for 6 months, enabling co-cultures with different cell types and testing of bone anabolic drugs. Their bulk RNAseq data revealed the marker trajectory of ossification and human primary osteocyte formation *in vitro* over an initial 8-week period. Vitamin D3 supplementation enhanced mineralization and sclerostin release, while hypoxia and PTH1-34 affected sclerostin levels. FGF23 can be also detected in the culture media deriving from the realized culture system.

Evaluation of the effect of other cell populations on bone cell activity was also explored in a perspective article by Little-Letsinger and Hamilton. The study of bone-immune cell interaction can employ a particular animal model: the dirty mice. These mice, normally exposed to commensal and pathogenic microbes, show mature immune systems comparable to adult humans, distinct to the naïve immune system of specific-pathogen free mice similar to that of neonates. Investigation of the dirty mouse model will be useful in studying diseases with an interplay between overactivation of the immune system and negative bone outcomes, such as osteoporosis, fractures, arthritis, and bone cancers.

Corrao et al. with The Italian Fragility Fracture Team wrote a systematic review to delineate the Italian guidelines for diagnosis, risk stratification, and care continuity of fragility fractures 2021. They addressed a very important question because fragility fractures represent a major public health concern owing to their growing burden and their onerous costs for the health systems. The reported guidelines aim to provide evidence-based recommendations for recognizing, risk stratifying, treating, and managing patients with fragility fracture. The authors recommended recognition of bone fragility as the key cause of fractures, measurement of individual (re)fracture risk using a validated tool, assessment of exposure to different factors correlated with potential (re)fracture risk, use of a sequential pharmacologic scheme from anabolic to anti-resorptive drugs, primarily in patients at higher risk of fracture, avoidance of treatment interruption, and increased multidisciplinary care systems in order to ensure patients’ transition to hospital outpatient services. These guidelines will support individualized management of patients experiencing non-traumatic bone fracture, helping in secondary prevention of (re)fracture.

Otani et al. in a mini-review reported the cellular mechanisms of osteosarcoma development. Osteosarcoma is a primary malignant bone tumor. The review describes the different experimental conditions and new technological approaches leading to the identification of the osteosarcoma cancer cell, known to be in the osteoblastic lineage. Stem cells within the bone marrow endoskeletal region readily generated osteosarcoma cells mainly under p53 deletion conditions. This review concluded with a discussion of future approaches in the study of skeletal cells and osteosarcoma.

In conclusion, all together these papers highlighted different aspects of bone research in 2022.

## Author contributions

GB: Writing – original draft. JT: Writing – review & editing.
